# The effects of short video app–guided mindfulness meditation on rumination, self-compassion, psychological flexibility, and depression among individuals experiencing entrepreneurial failure

**DOI:** 10.3389/fpsyt.2025.1706661

**Published:** 2025-12-03

**Authors:** Ye-hui Tu, Chao Liu, Zheng-Hao Gao, Hao Chen

**Affiliations:** 1 School of Journalism and Communication, Hua Qiao University, Xiamen, China; 2 Business Analytics Research Center, Chang Gung University, Taoyuan, Taiwan; 3 Mindfulness Meditation Center, Ming Chi University of Technology, New Taipei, Taiwan; 4 School of Film Television & Communication, Xiamen University of Technology, Xiamen, China

**Keywords:** mindfulness meditation, rumination, self-compassion, psychological flexibility, depression

## Abstract

**Objective:**

This study aimed to evaluate the effectiveness of a short video app–guided mindfulness meditation intervention in improving psychological flexibility and self-compassion and reducing rumination and depressive symptoms among failed entrepreneurs.

**Methods:**

A randomized controlled trial was conducted with 100 recently failed entrepreneurs (aged 25–55), who were randomly assigned to either an intervention group (n = 50) or a waitlist control group (n = 50). Over five weeks, the intervention group completed daily 3-minute guided mindfulness meditation sessions via a secure mobile app. Psychological outcomes—including rumination, self-compassion, psychological flexibility, and depression—were assessed at pre- and post-intervention using validated instruments(Self-Compassion Scale – Short Form, SCS-SF), psychological flexibility (Acceptance and Action Questionnaire–II, AAQ-II). Data were analyzed using a two-step approach: a 2 (Group: Intervention vs. Control) × 2 (Time: Pre vs. Post) mixed-design MANOVA was first conducted to assess overall multivariate effects, followed by repeated measures ANOVAs for each outcome variable.

**Results:**

Significant group × time interaction effects were observed across all outcome variables. Compared to the control group, the intervention group showed significant reductions in rumination (*F* (1, 98) = 14.485, *p* <.001, η² = .073) and depression (*F*(1, 98) = 9.241, *p* = .003, η² = .045), as well as significant improvements in self-compassion (*F*(1, 98) = 11.764, *p* <.001, η² = .057) and psychological flexibility (*F*(1, 98) = 13.464, *p* <.001, η² = .064).

**Conclusions:**

This study provides robust empirical support for the efficacy of mobile-guided mindfulness interventions in promoting mental health recovery among failed entrepreneurs. The findings highlight the potential of short video–based mindfulness practices as scalable, low-barrier tools to reduce cognitive vulnerability and emotional distress following entrepreneurial failure.

**Clinical trial registration:**

Chinese Clinical Trial Registry (ChiCTR), identifier ChiCTR2500106524.

## 1 Introduction

In recent years, entrepreneurship has been widely promoted as a pathway to innovation, employment, and economic vitality. However, the harsh reality is that a significant proportion of start-up ventures end in failure. For entrepreneurs, such failures are not merely financial losses but also profound psychological setbacks. Previous studies have shown that failed entrepreneurs often suffer from persistent rumination, depressive symptoms, a lack of psychological flexibility, and diminished self-compassion, all of which negatively affect their well-being and hinder future entrepreneurial engagement (1, 2).

While conventional interventions such as psychotherapy or peer counseling are valuable, they often face barriers such as cost, accessibility, and social stigma. In response, there has been growing interest in mindfulness-based interventions (MBIs), which emphasize non-judgmental awareness of the present moment and have demonstrated effectiveness in alleviating stress, anxiety, and depression (2, 3). Among various mindfulness practices, short-form digital mindfulness programs, especially those delivered via short video apps, offer a low-threshold and scalable option, making mental health support more accessible to time-constrained and resource-limited populations like failed entrepreneurs.

Despite the promise of mobile-guided mindfulness, few empirical studies have explored its impact specifically among entrepreneurs who have experienced failure. Moreover, existing research rarely integrates multiple psychological constructs—such as rumination, self-compassion, psychological flexibility, and depression—into one coherent framework to assess the comprehensive effectiveness of digital mindfulness training.

Compared to widely used commercial mindfulness apps such as *Calm* and *Headspace* (4, 5), this study introduces a short video app–guided mindfulness intervention that differs in several key ways. First, it targets a psychologically vulnerable and under-researched population—recently failed entrepreneurs—whose needs are seldom addressed in digital mental health research. Second, the intervention utilizes ultra-brief (3-minute) videos via a short-form video app interface familiar to participants (e.g., Douyin), enhancing usability and adherence among time-constrained individuals. Third, the content was tailored specifically to themes such as failure acceptance, self-forgiveness, and emotional adjustment post-entrepreneurial loss—topics not commonly addressed in generic mindfulness content. Finally, the intervention is grounded in an integrative cognitive-affective model of mindfulness-based emotion regulation, offering a more targeted pathway of psychological change compared to commercially available programs.

Therefore, the present study aims to examine the effects of short video app–guided mindfulness meditation on the mental health of failed entrepreneurs. Specifically, this study investigates whether such an intervention can significantly reduce rumination and depressive symptoms, while enhancing self-compassion and psychological flexibility. By leveraging a scalable and engaging digital platform, this study not only addresses a critical research gap but also offers practical implications for promoting psychological recovery and long-term reintegration for individuals affected by entrepreneurial failure.

## 2 Literature review

### 2.1 Mindfulness meditation and rumination

Rumination, defined as a repetitive focus on negative thoughts and emotions, is a core cognitive vulnerability for depression (6). Mindfulness meditation targets this maladaptive pattern by cultivating meta-awareness and decentering—two key mechanisms that interrupt automatic negative thinking loops.

Unlike suppression or avoidance, mindfulness teaches individuals to observe thoughts as transient mental events rather than accurate reflections of the self. This process reduces emotional reactivity and promotes cognitive flexibility (7). Empirical studies confirm that mindfulness-based interventions significantly reduce rumination. For example, Ramel et al. (2004) (8) found that an 5-week mindfulness program lowered rumination among individuals with residual depressive symptoms. Jain et al. (2007) (9) also demonstrated that mindfulness outperformed relaxation training in reducing ruminative tendencies.

In the context of failed entrepreneurs, who often experience persistent self-blame and cognitive fixation, mindfulness offers a promising, low-cost strategy for disrupting rumination and promoting emotional recovery.

### 2.2 Mindfulness meditation and self-compassion

Self-compassion involves treating oneself with kindness, recognizing shared humanity, and maintaining mindful awareness during times of suffering (10). It serves as a protective factor against shame, self-criticism, and depressive symptoms—common emotional responses following entrepreneurial failure.

Mindfulness meditation naturally cultivates self-compassion by fostering non-judgmental awareness and reducing self-identification with negative thoughts. As individuals learn to observe internal experiences without avoidance or aversion, they become more accepting of personal flaws and setbacks. This cognitive shift—from harsh self-blame to kind observation—represents a key mechanism by which mindfulness improves emotional well-being.

Research has shown that mindfulness training significantly increases self-compassion levels. For example, Shapiro et al. (2005) (11) found that participation in an 5-week mindfulness program led to notable improvements in self-compassion and reduced anxiety. Similarly, Birnie et al. (2010) (12) demonstrated that greater mindfulness practice frequency was positively correlated with self-compassion growth.

For failed entrepreneurs, who often struggle with internalized failure narratives, cultivating self-compassion through mindfulness may reduce psychological distress and support healthier self-concept restoration.

### 2.3 Mindfulness meditation and psychological flexibility

psychological flexibility refers to one’s capacity to recover from adversity, adapt to change, and maintain mental well-being despite stress or trauma. For failed entrepreneurs, enhancing psychological flexibility is critical for overcoming emotional setbacks and re-engaging with future opportunities.

Mindfulness meditation contributes to psychological flexibility by strengthening emotional regulation, stress tolerance, and cognitive flexibility. Through consistent practice, individuals learn to observe distressing experiences without immediate reaction or avoidance, allowing for more adaptive coping responses (13). This non-reactive stance enables individuals to process failure with greater composure and psychological stability.

Empirical evidence supports the role of mindfulness in fostering psychological flexibility. For instance, Hölzel et al. (2011) (14) found that mindfulness training enhances brain regions associated with self-regulation and adaptive coping. In a study of workplace stress, Glomb et al. (2011) (15) demonstrated that mindfulness interventions led to increased psychological flexibility and reduced burnout. Moreover, mindfulness has been shown to buffer the impact of stress by promoting present-moment awareness and acceptance (16).

For individuals navigating the emotional aftermath of business failure, mindfulness may serve as a vital psychological resource that fosters perseverance, adaptability, and long-term recovery.

### 2.4 Mindfulness meditation and depression

Depression is commonly associated with persistent negative affect, cognitive distortions, and diminished emotional regulation. In the aftermath of entrepreneurial failure, individuals may experience intense feelings of worthlessness, hopelessness, and withdrawal—all hallmark features of depression.

Mindfulness meditation has been shown to alleviate depressive symptoms by disrupting automatic negative thought patterns and enhancing present-moment emotional regulation. Unlike traditional approaches that aim to change thought content, mindfulness focuses on changing one’s relationship with thoughts and feelings—developing a stance of non-reactive, open awareness (17).

Numerous randomized controlled trials support the antidepressant effects of mindfulness. For instance, Teasdale et al. (2003) (7) found that mindfulness-based cognitive therapy (MBCT) significantly reduced relapse rates among individuals with recurrent depression. A meta-analysis by Hofmann et al. (2010) (18) further confirmed that mindfulness interventions yield moderate-to-large effect sizes in reducing depressive symptoms across clinical and non-clinical populations.

Given the emotional vulnerability of failed entrepreneurs, mindfulness may serve as a preventive and restorative strategy that reduces the intensity and duration of depressive episodes, offering a low-cost, accessible path to mental health recovery.

### 2.5 Theoretical integration of psychological constructs

The selection of the four psychological constructs—rumination, self-compassion, psychological flexibility, and depression—is grounded in mindfulness-based emotion regulation theory and cognitive-affective processing models. These variables represent interrelated stages in the psychological response to entrepreneurial failure and are theoretically linked through a dynamic process model of emotional self-regulation.

Specifically, rumination is conceptualized as a maladaptive cognitive response to failure, characterized by repetitive and self-critical thought patterns that sustain negative affect and hinder recovery (6). Self-compassion provides an emotional counterbalance to rumination by promoting kindness toward the self and reducing harsh self-judgment, thereby facilitating adaptive emotion regulation (10). psychological flexibility reflects the capacity to tolerate distress and flexibly adapt to adversity; it is both a consequence of reduced rumination and a product of enhanced self-compassion (14). Finally, depression is understood as a downstream clinical outcome that is shaped by the interplay among these factors, particularly when individuals lack the cognitive and emotional resources to effectively process failure (18).

Taken together, these constructs form an integrated mediation pathway grounded in the logic of mindfulness-based mechanisms:

Mindfulness practice → reduction in rumination → increase in self-compassion and psychological flexibility → decrease in depressive symptoms.

This sequential model enhances the theoretical coherence of the present study and provides a compelling rationale for the inclusion of all four variables as both outcomes and intermediate mechanisms of psychological change.

### 2.6 Theoretical framework and hypotheses

This study is grounded in the Mindfulness-Based Intervention (MBI) theoretical model, which posits that mindfulness meditation fosters adaptive psychological outcomes through core mechanisms such as decentering, non-judgmental awareness, and emotion regulation (14, 16). The framework integrates empirical findings across four key psychological variables—rumination, self-compassion, psychological flexibility, and depression—all of which are highly relevant to the psychological experiences of failed entrepreneurs.

Mindfulness meditation enables individuals to shift from reactive, judgmental processing to an open and accepting stance toward internal experiences. This shift helps reduce rumination, a maladaptive cognitive style characterized by repetitive negative thinking, by fostering metacognitive awareness and interrupting automatic thought loops (7). Simultaneously, mindfulness nurtures self-compassion by promoting self-kindness and non-identification with perceived personal failure (10), thereby improving emotional responses to adversity.

Furthermore, the cultivation of present-moment awareness and acceptance enhances psychological flexibility, allowing individuals to adapt more flexibly to stressful events (13). As these internal resources are strengthened, the overall intensity and persistence of depressive symptoms are expected to decline.

Based on this framework, the following hypotheses are proposed:

H1:Short video app–guided mindfulness meditation will significantly reduce rumination among failed entrepreneurs.
H2:Short video app–guided mindfulness meditation will significantly enhance self-compassion among failed entrepreneurs.
H3:Short video app–guided mindfulness meditation will significantly increase psychological flexibility among failed entrepreneurs.
H4:Short video app–guided mindfulness meditation will significantly reduce depressive symptoms among failed entrepreneurs.

This theoretical framework underscores the potential of brief, scalable mindfulness interventions to foster emotional recovery following entrepreneurial failure and contributes to the growing literature on digital mental health strategies.

## 3 Methods

### 3.1 Participants

Participants were recently failed entrepreneurs recruited via national startup forums, entrepreneurship networks, and online platforms such as WeChat, Douban, and Zhihu. Eligibility criteria required participants to have experienced a significant business failure (e.g., company closure, bankruptcy, investor withdrawal, or forced liquidation) within the past 24 months. To ensure adequate digital literacy and psychological stability, participants were required to (1) be aged between 25 and 55 years (2), own a smartphone with regular use of short-form video apps (e.g., Douyin/TikTok), and (3) report no history of psychiatric diagnoses.

A total of 110 participants were assessed for eligibility and met all inclusion criteria. After providing informed consent, all participants were randomized into the experimental group (n = 55) and the control group (n = 55). In the experimental group, three participants did not start the intervention and one was lost during the five-week period, resulting in 51 completing the post-assessment. In the control group, two participants did not start the intervention and two were lost during the intervention, leaving 51 who completed the post-assessment. One participant from each group provided unqualified responses and was excluded during data screening. Consequently, 50 participants in each group (N = 100) were included in the final analysis.

The final sample had a mean age of 39.2 years (SD = 5.8), with the majority clustered between 32 and 45 years, representing the typical age range of active early- to mid-career entrepreneurs in China. Gender distribution was relatively balanced, with 58% male (n = 58) and 42% female (n = 42). In terms of educational background, 70% held a bachelor’s degree and 30% had a master’s degree or higher. Participants came from diverse entrepreneurial sectors, including technology and internet (42%), retail and service (30%), manufacturing (19%), and other industries (9%). Notably, 95% of participants reported daily use of short-form video platforms, confirming the ecological validity of the app-based intervention method.

To ensure baseline equivalence, stratified randomization was conducted based on gender and age. **Table 1** provides a detailed breakdown of demographic characteristics across the two groups.

**Table 1:** Demographic characteristics of participants by Group (N = 100).

Variable	Mindfulness Group (n = 50)	Control Group (n = 50)	Total (N = 100)
Age (years)			
Mean (SD)	39.28 (4.4)	39.60 (5.36)	39.44 (4.90)
Range	32–51	31–52	31–52
Gender			
Male, n (%)	29 (58%)	29 (58%)	58 (58%)
Female, n (%)	21 (42%)	21 (42%)	42 (42%)
Education Level			
Bachelor’s degree	34 (68%)	36 (72%)	70 (70%)
Master’s or higher	16 (32%)	14 (28%)	30 (30%)
Previous Business Type			
Tech/Internet	22 (44%)	20 (40%)	42 (42%)
Retail/Service	14 (28%)	16 (32%)	30 (30%)
Manufacturing	9 (18%)	10 (20%)	19 (19%)
Other	5 (10%)	4 (8%)	9 (9%)
Short Video App Use			
Daily User	48 (96%)	47 (94%)	95 (95%)
Occasional User	2 (4%)	3 (6%)	5 (5%)

### 3.2 Intervention description

Participants in the intervention group received a 5-week structured mindfulness meditation program, delivered through a secure and dedicated mobile platform designed for smartphone-based learning. The intervention content was developed in collaboration with certified mindfulness trainers and formatted into daily short-form videos, each approximately 3 minutes in length (19, 20). These videos were designed to accommodate participants’ busy post-failure adjustment periods while maintaining the therapeutic dosage required for psychological change (21, 22).

Participants were randomly assigned to either the intervention or control group using a computer-generated randomization sequence prepared before recruitment. Allocation was performed by an independent research assistant who was not involved in intervention delivery or data analysis, ensuring allocation concealment. Due to the behavioral nature of the intervention, participant blinding was not feasible. However, all outcome scoring and statistical analyses were conducted by a researcher blinded to group assignment to minimize potential bias.

The five-week intervention duration was chosen based on a balance between empirical precedent and digital feasibility. While traditional mindfulness-based programs such as MBCT and MBSR span 8–10 weeks, recent research suggests that shorter-duration MBIs**—**especially those delivered via mobile or online platforms—can still yield meaningful psychological benefits (23, 24).

Moreover, pilot testing of the short-video format indicated that user engagement tended to decline after 5–6 weeks, suggesting that a five-week window may optimize adherence while maintaining therapeutic exposure. This aligns with emerging findings that brief, high-frequency micro-interventions may be more suitable for digital environments with attentional constraints (25). Thus, the five-week structure was designed to balance effectiveness with real-world feasibility.

The meditation videos included core mindfulness components such as breath awareness, body scanning, emotion observation, and self-acceptance, recorded in Mandarin and accompanied by neutral background audio. Each day’s session was thematically structured and followed a progressive framework (26):

Week 1: Grounding attention in the present moment (e.g., breath and body awareness)
Week 2: Observing thoughts and emotions without judgment
Week 3: Accepting unpleasant feelings and failures as part of experience
Week 4: Developing self-kindness and emotional balance
Week 5: Integrating mindfulness into everyday thinking and behavior

The mindfulness intervention materials were designed and recorded by two certified mindfulness instructors with extensive professional training. Both instructors hold internationally recognized MBSR (Mindfulness-Based Stress Reduction) teaching qualifications accredited by the University of Massachusetts Center for Mindfulness. Each has more than five years of experience conducting mindfulness and compassion-based programs for clinical and community populations in mainland China and Taiwan.

To ensure scientific validity and psychological safety, all video scripts were co-reviewed by a senior clinical psychologist (Ph.D. in Clinical Psychology) specializing in stress reduction and emotional regulation. The content was then refined collaboratively to ensure cultural appropriateness for individuals who had experienced entrepreneurial failure. The final videos included guided breathing, body awareness, and self-compassion exercises recorded in Mandarin with professional audio quality and visual guidance to facilitate immersive engagement.

The mindfulness intervention was delivered via a proprietary mobile application temporarily named MindEase, jointly developed by the research team and the University’s Human-Computer Interaction Laboratory. The platform was compatible with both Android and iOS systems and built using Flutter 3.7 with a Firebase backend. It supported high-quality video streaming, daily push notifications, adherence tracking, and in-app self-reflection logs.

All user data were encrypted both in transit and at rest using industry-standard protocols (TLS 1.3 and AES-256). The app complied with national data protection regulations and institutional ethical guidelines. No personally identifiable information was collected; instead, anonymized usage data (e.g., number of sessions completed, total practice time) were stored on secure, university-maintained cloud servers accessible only to authorized research personnel.

Each session began with a brief 20-second introduction explaining the focus of the day, followed by guided audio-video instructions, and concluded with a self-reflection prompt displayed on-screen, encouraging participants to record emotional or cognitive responses to the practice. Participants were encouraged (but not forced) to log brief written reflections after each session (27).

To support adherence, the app issued automated daily notifications at participants’ preferred time slots and recorded their session completion status. The platform automatically tracked key engagement metrics such as session count, completion rates, and cumulative practice time, which were later used in exploratory analyses to assess dose–response relationships (22).

Throughout the intervention period, participants also received weekly encouragement messages via email or in-app pop-ups. These messages reinforced core mindfulness concepts, addressed common barriers (e.g., boredom, avoidance), and provided motivational content tailored to failed entrepreneurs coping with emotional setbacks (28).

In accordance with ethical and safety protocols, participants’ responses to the suicidality item in the PHQ-9 (“thoughts that you would be better off dead or of hurting yourself”) were automatically screened via the survey platform. If a participant selected a response of 3 or higher (i.e., “more than half the days” or “nearly every day”), a system-triggered alert was generated and reviewed by a licensed psychological consultant. These participants were contacted within 24 hours and provided with referral information to crisis hotlines and mental health services.

All procedures, including the suicide risk monitoring protocol, were reviewed and approved by the Institutional Review Board of Chang Gung University (IRB No. 20250721B0). This IRB served as the sole ethical oversight body for the entire study, and the reference has been unified throughout the manuscript for consistency.

**Figure 1:**
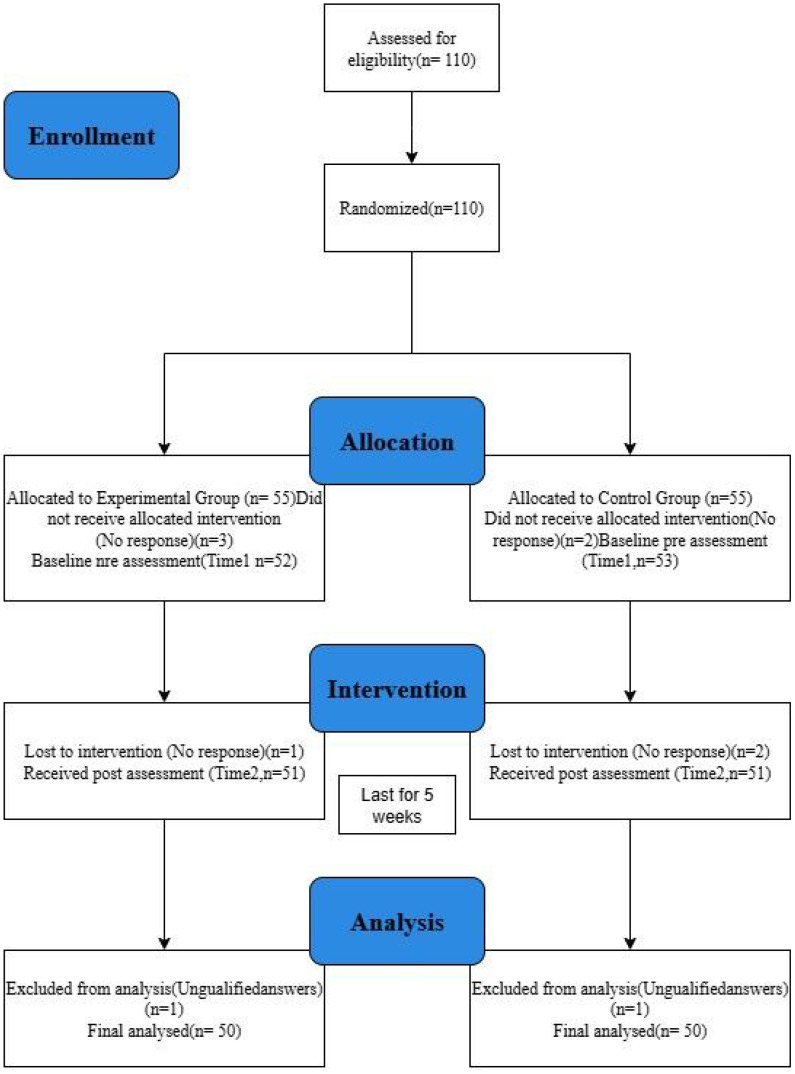
Procedure flow chart.

Participants in the control group received no psychological training or intervention during the study period but were informed that they would receive access to all mindfulness materials upon study completion, ensuring ethical transparency. The intervention procedure and weekly content structure are illustrated in **Figure 1**.

### 3.3 Measures

#### 3.3.1 Psychological flexibility

psychological flexibility was assessed using a modified version of the Acceptance and Action Questionnaire–II (AAQ-II), originally developed by Bond et al. (2011) (29). To ensure consistency across instruments, all items were adapted to a 5-point Likert scale ranging from 1 (strongly disagree) to 5 (strongly agree). The AAQ-II consists of 7 items measuring psychological inflexibility and experiential avoidance, which inversely reflect psychological flexibility. Higher scores indicate greater inflexibility and thus lower psychological flexibility. The AAQ-II has been validated across multiple populations and is commonly used in mindfulness and emotion regulation research. The original 7-point response scale of the AAQ-II was adapted to a 5-point Likert format (1 = strongly disagree, 5 = strongly agree) for consistency across all study instruments and to reduce cognitive load. A pilot test (n = 30) showed that the adapted version maintained good internal consistency (Cronbach’s α = 0.821).

Exploratory factor analysis using principal axis factoring yielded a single-factor solution(KMO = 0.921; Bartlett’s χ² = 602.650, df = 21, p < .001), explaining 59.08% of total variance, confirming the unidimensional structure consistent with the original scale.

#### 3.3.2 Depression

Depressive symptoms were measured using the Patient Health Questionnaire-9 (PHQ-9) (30), a widely used screening tool for depression. The original 4-point frequency scale was adapted to a 5-point Likert scale (1 = not at all, 5 = nearly every day) to match the scaling of other instruments in the study. The 9 items assess common symptoms of depression such as sadness, anhedonia, fatigue, sleep disturbance, and suicidal ideation. Higher scores reflect greater severity of depressive symptoms. This adapted version maintains the scale’s strong internal consistency and clinical relevance while improving cross-instrument comparability. The PHQ-9 response format was modified from the original 4-point to a 5-point Likert scale (1 = not at all, 5 = nearly every day) to ensure scale compatibility. The adapted version demonstrated strong internal reliability (Cronbach’s α = 0.858).Exploratory factor analysis supported a unidimensional structure (KMO = 0.933; Bartlett’s χ² = 763.928, df = 36, p < .001), explaining 54.14% of the total variance, consistent with the original validation of the PHQ-9.

The PHQ-9 response format was adapted from the original 4-point scale (0 = not at all, 3 = nearly every day) to a 5-point Likert scale (1 = not at all, 5 = nearly every day) to harmonize response ranges across all study instruments and optimize delivery via mobile devices. Although previous research has shown that increasing Likert granularity may improve psychometric sensitivity without compromising reliability (31, 32), the adapted format renders standard clinical thresholds (e.g., 5 = mild, 10 = moderate) inapplicable. As such, we interpreted PHQ-9 scores in terms of relative change rather than absolute severity classification.

#### 3.3.3 Self-compassion

Self-compassion was evaluated using the 12-item Self-Compassion Scale – Short Form (SCS-SF) (33), based on Neff’s (2003) (34) original scale. The instrument covers three core dimensions: self-kindness, common humanity, and mindfulness. Participants rated each item on a 5-point Likert scale (1 = almost never, 5 = almost always). Example item: “When I fail at something important, I try to keep things in perspective.” Higher scores indicate higher levels of self-compassion. The SCS-SF has been shown to have good convergent validity with well-being and emotional regulation measures(Cronbach’s α = 0.894).

#### 3.3.4 Rumination

Rumination was assessed using a 6-item short form of the Ruminative Responses Scale (RRS), revised by Treynor, Gonzalez, and Nolen-Hoeksema (2003) (35). This version includes two subdimensions: brooding and reflection, with three items each. All items were rated on a 5-point Likert scale (1 = almost never, 5 = almost always). Brooding reflects passive dwelling on distress, whereas reflection indicates purposeful introspection. Higher scores denote greater rumination tendencies. The short-form RRS has demonstrated strong psychometric properties and is frequently used in studies of cognitive vulnerability to depression.(Cronbach’s α = 0.774).

The Chinese version of the RRS used in this study includes two validated subdimensions: brooding and reflection (35). However, we computed and analyzed the total RRS score to capture participants’ overall tendency toward ruminative thinking following entrepreneurial failure. This approach was selected to reflect global cognitive reactivity, rather than process-specific mechanisms, which aligns with previous mindfulness-based intervention research (36). While the distinction between brooding and reflection is theoretically meaningful, it was beyond the scope of this study. We acknowledge this as a limitation and recommend that future studies examine subscale-level effects to better understand differential pathways of change.

All instruments originated from validated English-language scales published in peer-reviewed journals. Since participants were Chinese-speaking failed entrepreneurs, we employed a rigorous two-way translation process to ensure conceptual and linguistic accuracy. A bilingual Ph.D. researcher first translated the scales into Chinese. An English-speaking master’s student independently translated them back into English. This back-translation process followed Brislin’s (1980) (37) guidelines to maintain semantic equivalence.

Three bilingual experts in health psychology and communication reviewed both language versions for clarity and cultural relevance. We resolved discrepancies through discussion. Before the main study, we conducted a pilot test with 30 Chinese mid-level failed entrepreneurs to assess item clarity and cultural appropriateness. Based on participant feedback, we made minor revisions to enhance readability.

### 3.4 Procedure

This study followed a two-group, pretest–posttest randomized controlled design conducted over a period of five weeks. The procedure consisted of five key stages: recruitment, screening, baseline assessment, intervention, and post-intervention assessment.

#### 3.4.1 Recruitment and screening

Participants were recruited using convenience sampling through national entrepreneurship forums, startup support organizations, and social media platforms such as WeChat, Zhihu, and Douban. Interested individuals were first screened via an online questionnaire to determine eligibility, which included recent entrepreneurial failure (within the past 24 months), smartphone ownership, and regular use of short video apps. Those meeting inclusion criteria were invited to provide informed consent and complete the baseline assessment.

#### 3.4.2 Randomization

A total of 100 eligible participants were randomly assigned to either the mindfulness intervention group (n = 50) or the waitlist control group (n = 50), using stratified randomization based on gender and age to ensure group equivalence at baseline.

#### 3.4.3 Baseline assessment

Before the intervention, all participants completed a battery of standardized psychological instruments measuring rumination, self-compassion, psychological flexibility, and depression, via a secure online survey platform. To ensure translation fidelity, all instruments were adapted through Brislin’s (1980) back-translation method.

#### 3.4.4 Intervention implementation

Participants in the intervention group received access to a custom-designed mobile application delivering daily short-form guided mindfulness meditation videos (see Section 3.2). The app logged user engagement and sent daily reminders. Participants in the control group received no treatment during the five-week period but were promised post-study access to all materials. The intervention flow is illustrated in **Figure 1**.

#### 3.4.5 Post-intervention assessment

At the conclusion of the five-week period, all participants completed the same psychological measures administered at baseline. In addition to quantitative data, participants in the intervention group were asked to provide qualitative feedback on their experience with the app and meditation content.

All procedures were approved by the institutional ethics committee, and all participants were informed of their right to withdraw at any time without penalty.

### 3.5 Statistical analysis

All statistical analyses were conducted using IBM SPSS Statistics (Version 26.0). To evaluate the overall effects of the intervention on the combined set of psychological outcomes (rumination, self-compassion, psychological flexibility, and depression), we first conducted a 2 (Group: Intervention vs. Control) × 2 (Time: Pre vs. Post) mixed-design MANOVA. This multivariate approach allowed us to assess whether the mindfulness intervention had a statistically significant multivariate effect across all outcome variables collectively.

Following a significant Group × Time interaction in the MANOVA, we proceeded with a series of follow-up repeated measures ANOVAs (RM-ANOVAs) for each dependent variable to examine individual effects. Interaction effects between group and time were tested for each outcome, and partial eta squared (η²) was reported as an effect size index.

Data were analyzed following a per-protocol approach, including only participants who completed both pre- and post-assessments. Given the exploratory nature of this study and the small sample size, intention-to-treat (ITT) analyses were not performed, as imputing missing values could increase estimation error. Moreover, attrition occurred mainly before intervention commencement, not during the active phase, making the per-protocol analysis more appropriate for assessing the actual effects of the intervention among engaged participants.

## 4 Result

In this study, a two-arm randomized controlled trial design of 2 (Group: Mindfulness, Control) × 2 (Time: Pre, Post) was adopted, and MANOVA was used to examine the differential effects of the intervention on participants’ scores on the RRS (Ruminative Responses Scale), AAQ-II (Acceptance and Action Questionnaire–II), SCS-SF (Self-Compassion Scale – Short Form), and PHQ-9 (Patient Health Questionnaire–9).

Among the 50 participants in the intervention group, the mean number of completed sessions was 33.5 out of 35 (SD = 2.2). A total of 92% (n = 46) completed at least 80% of the intervention sessions, demonstrating strong adherence to the mindfulness protocol. These results indicate high feasibility and acceptability of the short video–based delivery format.

Results of Multivariate Tests showed that there was a significant main effect on the Group: F (4, 193)=10.869, p<0.001, η2 = 0.184; a significant main effect on Time: F (4, 193)=11.309 p<0.001, η2 = 0.190; and a significant interaction effect between Group and Time: F (4, 193)=10.689, p<0.001, η2 = 0.181.**Table 2** presents the descriptive statistics of all outcome variables across groups and timepoints.

**Table 2:** Descriptive statistics and pre–post comparison by Group (Rumination, Self-Compassion, psychological flexibility, and Depression).

Group	Measures	Mean (SD)	
		Pre	Post
MM (n=50)	AAQ-II	24.08(5.886)	18.26(4.193)
	RRS	20.08(4.304)	16.20(3.774)
	SCS-SF	28.72(5.718)	35.96(8.559)
	PHQ-9	32.92(7.631)	26.86(5.792)
Control (n=50)	AAQ-II	24.16(2.108)	23.92(6.107)
	RRS	20.22(3.334)	20.40(3.051)
	SCS-SF	29.00(6.960)	29.24(7.339)
	PHQ-9	32.70(6.402)	32.34(6.558)

SCS-SF, Self-Compassion Scale–Short Form; AAQ-II, Acceptance and Action Questionnaire–II; RRS, Ruminative Responses Scale; PHQ-9, Patient Health Questionnaire–9.Higher scores reflect greater levels of self-compassion (SCS-SF), rumination (RRS), and depression (PHQ-9). Lower scores on the AAQ-II indicate greater psychological flexibility and flexibility.

**Figure 2:**
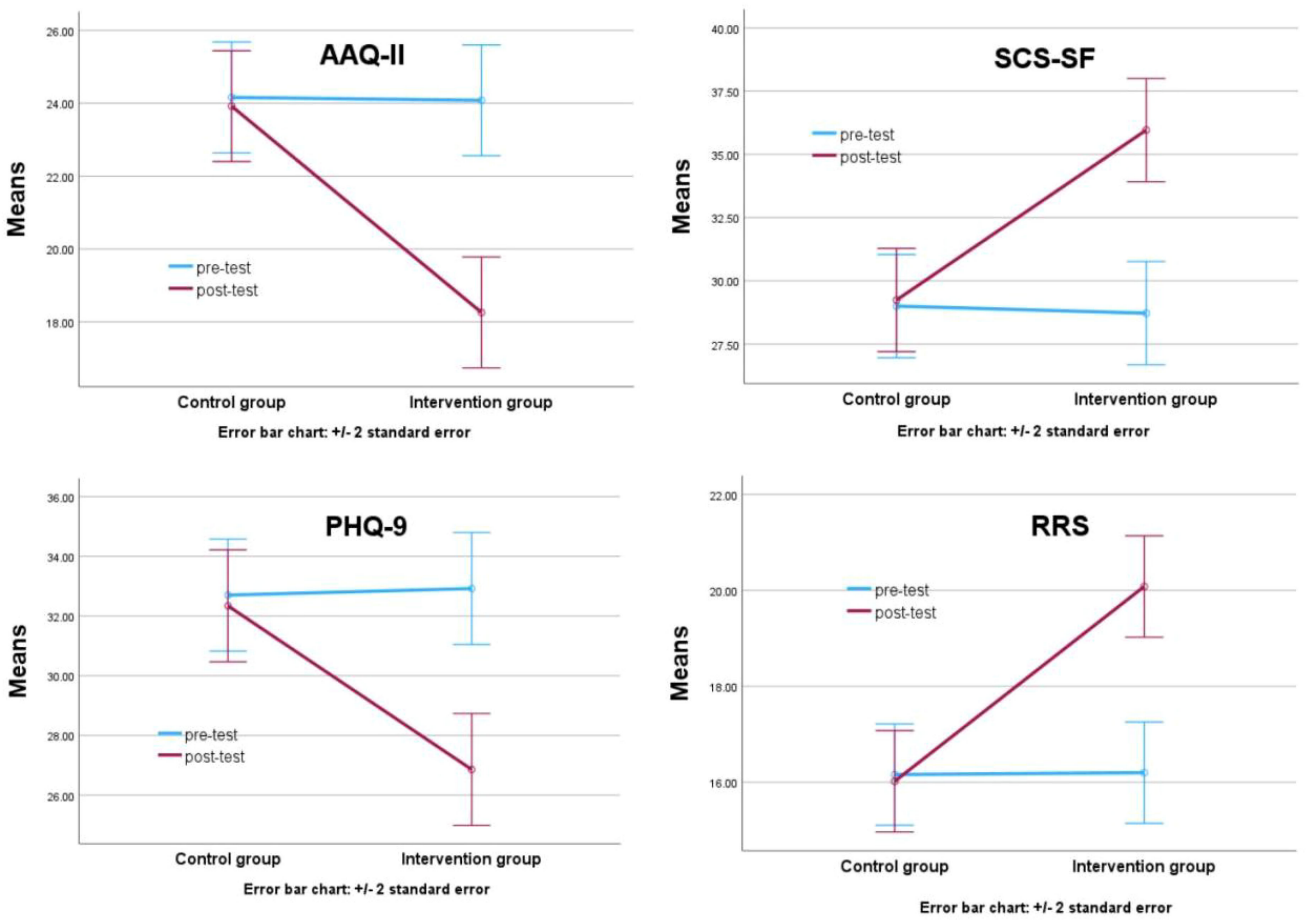
Significant interaction effects between group and time. AAQ-II, the Acceptance and Action Questionnaire–II; RRS, the Ruminative Responses Scale; PHQ-9, the Patient Health Questionnaire-9; SCS-SF, Self-Compassion Scale – Short Form.

The results of **Table 3** reveal significant interaction effects between Group and Time across the four outcome measures: RRS (Rumination Response Scale), SCS-SF (Self-Compassion Scale – Short Form), AAQ-II (Acceptance and Action Questionnaire-II), and PHQ-9 (Patient Health Questionnaire-9). **Figure 2** illustrates these interaction effects in greater detail.

**Table 3:** Repeated measures ANOVA results for Group × Time interaction effects and effect sizes (Partial η²).

Measures	ariable	F	p	Partial eta squared (η²)
AAQ-II	Group^***^	14.247	<0.001	0.068
	Time^***^	15.880	<0.001	0.075
	Group×Time^***^	13.464	<0.001	0.064
RRS	Group^***^	17.706	<0.001	0.083
	Time^***^	12.869	<0.001	0.062
	Group×Time^***^	15.495	<0.001	0.073
SCS-SF	Group^**^	9.957	0.002	0.048
	Time^**^	13.432	<0.001	0.064
	Group×Time^**^	11.764	<0.001	0.057
PHQ-9	Group^**^	7.870	0.006	0.039
	Time^***^	11.724	<0.001	0.056
	Group×Time^**^	9.241	0.003	0.045

AAQ-II, the Acceptance and Action Questionnaire–II; RRS, the Ruminative Responses Scale; PHQ-9, the Patient Health Questionnaire-9; SCS-SF, Self-Compassion Scale – Short Form. AAQ-II, Acceptance and Action Questionnaire–II; lower scores indicate better psychological flexibility.*p < 0.05; **p < 0.01; ***p < 0.001. The colored values indicate statistically significant results (*p < 0.05, **p < 0.01, *p < 0.001).

A 2 (Group: Mindfulness vs. Control) × 2 (Time: Pre vs. Post) mixed-design MANOVA was conducted to examine the effects of the short video app–guided mindfulness meditation intervention on four psychological outcomes: rumination, self-compassion, psychological flexibility, and depression. The results revealed a significant Group × Time interaction effect, indicating that the intervention produced differential changes across the two groups.

Among the 50 participants in the intervention group, the mean number of completed sessions was 33.5 out of 35 (SD = 2.2). Notably, 46 participants (92%) completed at least 80% of the sessions, indicating strong adherence to the program. These engagement metrics suggest high feasibility and acceptability of short-video–based mindfulness practice among failed entrepreneurs.

Effect sizes were reported as partial eta squared (η²). Following Cohen’s (2013) (38) conventions, η² values of.01,.06, and.14 represent small, medium, and large effects, respectively. This classification provides a basis for interpreting the magnitude and practical significance of the observed intervention effects.

### 4.1 Rumination

As shown in **Table 3**, participants in the intervention group exhibited a significant reduction in rumination, as measured by the Ruminative Responses Scale (RRS). Their mean scores decreased from M = 20.08 (SD = 4.304) at pre-test to M = 16.20 (SD = 3.774) at post-test. In contrast, the control group showed no notable change (Pre: M = 20.22, SD = 3.334; Post: M = 20.40, SD = 3.051). A repeated measures ANOVA revealed a significant Group × Time interaction, *F* (1, 98) = 14.485, *p* <.001, η² = .073, indicating that the mindfulness intervention was effective in reducing maladaptive repetitive thinking patterns.

According to Cohen’s guidelines, the effect size (η² = .073) represents a moderate effect, indicating that the intervention had a practically meaningful impact on reducing participants’ rumination levels.

This pattern of change provides empirical support for Hypothesis 1 (H1), which proposed that the intervention would significantly reduce levels of rumination.

### 4.2 Self-compassion

For self-compassion, measured using the SCS-SF, a substantial improvement was observed in the intervention group, with mean scores increasing from M = 28.72 (SD = 5.718) to M = 35.96 (SD = 8.559). In contrast, the control group remained relatively unchanged (Pre: M = 29.00, SD = 6.960; Post: M = 29.24, SD = 7.339). The Group × Time interaction was statistically significant, *F* (1, 98) = 11.764, *p* <.001, η² = .057, suggesting that the brief mindfulness practice was effective in cultivating greater self-directed compassion among failed entrepreneurs.

The observed effect size (η² = .057) is slightly below the conventional threshold for a medium effect but above the threshold for a small effect. It can be interpreted as a small-to-moderate effect, suggesting a noticeable improvement in self-compassion.

These findings are consistent with Hypothesis 2 (H2), which anticipated an increase in self-compassion as a result of the intervention.

### 4.3 Psychological flexibility

psychological flexibility was assessed using the Acceptance and Action Questionnaire–II (AAQ-II), where lower scores indicate greater flexibility and psychological flexibility. Participants in the intervention group showed a clear improvement, with scores decreasing from M = 24.08 (SD = 5.886) to M = 18.26 (SD = 4.193) post-intervention. In comparison, the control group’s scores remained stable (Pre: M = 24.16, SD = 2.108; Post: M = 23.92, SD = 6.107). The Group × Time interaction was significant, *F*(1, 98) = 13.464, *p* <.001, η² = .064.

The effect size for psychological flexibility (η² = .064) qualifies as a moderate effect, indicating that the mindfulness intervention meaningfully enhanced participants’ psychological flexibility and psychological flexibility.

These results confirm Hypothesis 3 (H3), which posited that the mindfulness intervention would increase psychological flexibility by enhancing acceptance and reducing experiential avoidance.

### 4.4 Depression (PHQ-9)

Lastly, the intervention also had a meaningful impact on depressive symptoms, as measured by the PHQ-9. The intervention group’s scores dropped from M = 32.92 (SD = 7.631) to M = 26.86 (SD = 5.792), while the control group showed only marginal change (Pre: M = 32.70, SD = 6.402; Post: M = 32.34, SD = 6.558). The interaction effect was statistically significant, *F*(1, 98) = 9.241, *p* = .003, η² = .045, indicating that the intervention group experienced greater relief from depressive symptoms over time.

The interaction effect size (η² = .045) falls within the small-to-moderate range, implying that while the change in depression scores was statistically significant, the magnitude of change was modest. Additionally, an exploratory correlation analysis revealed that the reduction in rumination (RRS) scores was significantly associated with a reduction in depressive symptoms (PHQ-9), r = .42, p <.001. This suggests that improvements in cognitive processing may contribute meaningfully to emotional recovery.

This outcome supports Hypothesis 4 (H4), which predicted a significant reduction in depression following mindfulness intervention.

Descriptive statistics for all outcome variables at both timepoints are presented in **Table 3**, and the Group × Time interaction trends are visually illustrated in **Figure 2**. These findings provide robust empirical support for the effectiveness of short video app–guided mindfulness meditation in improving the mental health of failed entrepreneurs. Specifically, the intervention significantly reduced rumination and depression, while enhancing self-compassion and psychological flexibility. The results also underscore the potential of scalable digital mindfulness programs in supporting emotional recovery and psychological flexibility-building in post-failure entrepreneurial contexts.

## 5 Discussion

This study investigated the effects of a short video app–guided mindfulness meditation intervention on four key psychological constructs—rumination, self-compassion, psychological flexibility, and depression—among failed entrepreneurs. The results revealed significant group × time interaction effects on all four outcome variables, supporting the hypothesis that brief, digitally delivered mindfulness interventions can promote psychological recovery and emotional self-regulation in high-stress populations. The following sections explore the possible mechanisms behind these findings.

### 5.1 Rumination: mindfulness as a mechanism of meta-cognitive regulation

The observed reduction in rumination suggests that mindfulness training may enhance meta-cognitive decentering—a core mechanism in mindfulness-based cognitive therapy (MBCT)—enabling individuals to observe negative thoughts without identifying with them. This perspective aligns with Teasdale et al. (2003) (7), who proposed that increased awareness disrupts the habitual activation of depressive thought patterns. The intervention likely enabled participants to recognize cognitive loops without engaging in brooding, thus breaking the cycle of negative self-focus that commonly follows entrepreneurial failure.

### 5.2 Self-compassion: reconstructing entrepreneurial identity after failure

The increase in self-compassion can be interpreted within Carson (2011) (39) Compassion-Focused Therapy framework, which emphasizes the transformation of inner dialogues from shame-based self-attack to soothing self-acceptance. Entrepreneurial failure often triggers intense self-blame and identity devaluation. The intervention’s repetition of compassionate phrases may have facilitated a shift from evaluative to affiliative processing, helping participants reinterpret their failure with greater kindness and contextual understanding.

### 5.3 Psychological flexibility: from inflexibility to values-based adaptation

The observed improvement in psychological flexibility—as indicated by lower AAQ-II scores—suggests that mindfulness training may reduce experiential avoidance and promote acceptance-based coping. This aligns with the theoretical foundation of Acceptance and Commitment Therapy (ACT), which emphasizes the cultivation of psychological flexibility as a key mechanism of mental health (40). Participants likely learned to relate differently to distressing internal experiences (e.g., shame, fear of failure) by observing them non-reactively rather than suppressing them, thus enabling more values-consistent behavior in the aftermath of failure.

Unlike traditional emotion suppression strategies often observed in entrepreneurs managing reputational damage, mindfulness invites an open stance toward suffering, allowing discomfort to coexist with purposeful action (40, 41). This interpretive shift—from control to willingness—may have played a central role in restoring adaptive functioning and goal re-engagement, two hallmarks of psychological flexibility. The app-based daily meditation likely created micro-moments of exposure to internal discomfort, serving as practice grounds for developing these adaptive responses (42).

### 5.4 Depression: rewiring the affective-cognitive loop

The significant reduction in depressive symptoms is likely the cumulative result of multiple interacting mechanisms facilitated by mindfulness meditation. As outlined in the emotion regulation model of mindfulness (43), enhanced attention regulation, increased emotional awareness, and reduced cognitive elaboration can work together to buffer depressive cascades. In our study, the observed reductions in rumination and increases in self-compassion likely contributed synergistically to reduced affective burden. Indeed, our data showed that changes in rumination and depression were moderately correlated (r = .42, p <.001), providing additional empirical support for this pathway.

Mindfulness may have also improved emotional granularity—the ability to differentiate emotional experiences with precision—which has been linked to better emotion regulation and lower depression risk. For failed entrepreneurs, who often experience self-critical, globalized negative affect, this capacity may reduce the perceived overwhelm of failure-related emotions.

Additionally, consistent with the decentering hypothesis, participants may have learned to see negative thoughts (e.g., “I am a failure”) as transient mental events rather than absolute truths, thus weakening their emotional impact. Although the brief format of short-video–guided meditation may seem limited, the structure likely facilitated high-frequency, low-barrier practice, which is increasingly recognized as effective for mood regulation.

### 5.5 Interaction effects and mechanisms of change

The significant Group × Time interaction effects across all variables provide strong evidence that psychological changes were driven by the mindfulness intervention rather than natural recovery or statistical regression. These effects likely emerged through a synergistic mechanism involving increased present-moment awareness, decentering from cognitive patterns, emotional acceptance, and self-soothing. The mobile delivery model may also have enhanced engagement through convenience and low emotional barrier to entry, making it easier for participants to internalize the techniques.

### 5.6 Short-video format as a scalable alternative to traditional digital MBIs

Compared with prior app- and web-based mindfulness-based interventions (MBIs), which typically yield small to moderate effects on depression and anxiety (23), our observed effects on rumination and psychological flexibility (η² = .073 and.064, respectively) fall within a comparable range. A distinctive feature of our study is the use of a short-video format, which may enhance engagement and lower participation barriers by aligning with users’ habitual content consumption patterns. This delivery method may also reduce drop-out, a common issue in digital MBIs (44), by offering more accessible and frequent micro-practice opportunities.

### 5.7 Policy and practice implications

This study highlights the potential of short video–guided mindfulness as a scalable, low-cost tool to support entrepreneurs recovering from failure—a group often neglected by traditional mental health services. Popular platforms like TikTok or Douyin could be adapted for structured micro-interventions that fit users’ daily media habits (45).

Governments and incubators could integrate such tools into post-failure recovery programs to promote psychological flexibility and reduce entrepreneurial attrition (46). More broadly, this format aligns with digital well-being goals, showing that meaningful psychological change is possible even in short-form, attention-limited environments (47).

Future research should examine long-term outcomes and whether this approach applies to other high-stress populations.

### 5.8 Cultural interpretation of self-compassion in Chinese contexts

In Chinese cultural contexts, self-compassion and mindfulness may be interpreted differently from Western norms. Influenced by Confucian ethics and dialectical thinking, Chinese individuals often value self-discipline and constructive self-criticism over overt self-kindness. This cultural framing may affect how participants internalize mindfulness practices—shifting from harsh self-judgment to balanced self-regulation rather than unconditional self-acceptance (48, 49). Our findings suggest that the observed increases in self-compassion may reflect culturally congruent emotional reframing rather than direct adoption of Western self-soothing narratives.

### 5.9 Limitations

Several limitations should be noted. First, the study focused solely on failed entrepreneurs in China, which may limit the generalizability of the findings to other populations or cultural contexts. Previous research has highlighted that cultural norms significantly influence how mindfulness and self-compassion practices are internalized and expressed, suggesting that intervention outcomes may differ across societies (50).

Second, reliance on self-report measures introduces potential bias due to social desirability or limited self-awareness. This concern is common in psychological studies and may be mitigated by combining self-reports with objective indicators or third-party assessments in future research (51).

Third, the intervention duration was relatively short (five weeks), which may not be sufficient for long-term psychological restructuring or sustained behavioral change. Moreover, no follow-up data were collected, preventing assessment of the durability of intervention effects over time (52).

Fourth, although we collected detailed adherence data such as daily session completion and total practice time via the app platform, we did not statistically examine the relationship between adherence and psychological outcomes. This omission limits our ability to assess potential dose–response effects and explore how varying levels of engagement may influence intervention efficacy.

Fifth, although the current study focused on immediate pre-post effects, future research may incorporate longer-term follow-up to evaluate the durability of intervention gains. Due to the behavioral nature of the intervention, participant blinding was not feasible; however, outcome assessment and data analysis were conducted by a researcher blinded to group allocation to reduce bias. Finally, while session adherence data were collected via the app, more comprehensive fidelity checks (e.g., real-time usage monitoring, qualitative feedback) could further illuminate engagement patterns and help explain variability in outcomes.

Sixth, the use of a waitlist control group may have introduced expectancy-related bias or nonspecific treatment effects. Although we did not assess participant expectations directly, future studies should consider using credibility ratings or active controls to isolate intervention-specific effects.

Seventh, while exploratory factor analysis supported the unidimensional structure of the adapted PHQ-9 and AAQ-II, future studies should formally assess longitudinal measurement invariance (configural, metric, scalar) across groups and timepoints to ensure construct equivalence and interpretability of change scores.

Eighth, although basic information regarding business sector and timing of the failure was collected (i.e., within the past 24 months), we did not obtain detailed measures of perceived severity, financial loss, or prior entrepreneurial failures, in order to protect participant anonymity and reduce disclosure burden. These considerations limited our ability to conduct moderation analyses based on failure characteristics.

Finally, the absence of objective behavioral or physiological data (e.g., heart rate variability, facial expressions, or actigraphy) restricts the understanding of underlying mechanisms. Multimodal data could provide richer insight into the embodied effects of mindfulness and better validate psychological outcomes (53).

### 5.10 Future research directions

Future studies should adopt a more diverse sample across regions, industries, and gender identities to enhance external validity and cultural representativeness (54). Researchers are also encouraged to incorporate multi-source data, such as peer ratings, behavioral logs, or physiological measures, to mitigate bias and enhance the triangulation of psychological constructs (55).

Extending the intervention period and including longitudinal follow-up measurements would help examine the sustainability and decay curves of the observed effects (56). Additionally, comparing mindfulness meditation with alternative interventions such as cognitive behavioral coaching, compassion-focused therapy, or acceptance-based approaches may reveal unique and shared mechanisms of psychological chang (57).

Cross-cultural comparative studies could further clarify how digital contemplative interventions function within different sociocultural frameworks, which is especially relevant in global entrepreneurship and mental health applications (58).

Future studies should include adherence metrics—such as frequency, duration, and consistency of use—as moderator or mediator variables to assess their predictive value on outcome changes. By doing so, researchers can better understand whether increased engagement leads to stronger psychological benefits and optimize mobile mindfulness interventions accordingly.

Additionally, the current analysis did not include confidence intervals for the main effects. Future studies should report confidence intervals to provide greater precision and interpretability of intervention effects.

Future studies should incorporate fidelity monitoring and engagement metrics (e.g., app usage logs, qualitative feedback) to better understand the mechanisms driving intervention efficacy.

Future research should incorporate follow-up assessments, monitor adherence through engagement metrics, and consider fidelity checks. Active control conditions may also help reduce expectancy effects and strengthen causal interpretation.

Future studies should consider conducting sensitivity analyses using standardized scores to verify the robustness and generalizability of findings across different scoring systems.(1.4).

Future studies may explore dose–response relationships between the number of completed sessions and psychological outcomes to better understand intervention efficacy and optimize implementation strategies.

Future studies with larger sample sizes and multiple timepoints should explore potential mediating mechanisms—such as whether changes in rumination and self-compassion account for reductions in depression—using structural modeling and bias-corrected bootstrapping procedures.

## 6 Conclusion

This study provides empirical evidence that a short video app–guided mindfulness meditation intervention can significantly improve the mental health of failed entrepreneurs. Specifically, participants who engaged in a five-week mobile mindfulness program exhibited reduced rumination and depressive symptoms, as well as increased self-compassion and psychological flexibility, compared to those in the control group. These findings confirm the effectiveness of delivering contemplative practices through scalable, low-barrier digital platforms, addressing a critical psychological need in a vulnerable yet often overlooked population. By integrating cognitive, emotional, and behavioral mechanisms of change, this study highlights the transformative potential of brief, tech-mediated mindfulness interventions in facilitating emotional recovery and psychological reintegration after entrepreneurial failure. Future research is encouraged to build on these insights using more diverse samples, longitudinal designs, and multi-method assessments to strengthen the generalizability and robustness of these outcomes.

## Data Availability

The original contributions presented in the study are included in the article/supplementary material. Further inquiries can be directed to the corresponding author.
